# An association between adropin hormone and total testosterone in obese men: a case-control study

**DOI:** 10.1186/s12902-022-01102-7

**Published:** 2022-07-27

**Authors:** Asmaa A. Muhammed, Rania M. H. M. Eid, Wafaa Salah Mohammed, Mahmoud R. Abdel-Fadeil

**Affiliations:** 1 Departments of Medical Physiology, Faculty of Medicine, Aswan University, Aswan, 81511 Egypt; 2 Department of Clinical Pathology, Faculty of Medicine, Aswan University, Aswan, Egypt; 3 Departments of Medical Physiology, Faculty of Medicine, Assiut University, Assiut, Egypt

**Keywords:** Adropin, Adiponectin, Total testosterone, Obesity

## Abstract

**Background:**

Obesity is associated with low testosterone levels that could be caused by many mechanisms. Adropin, a peptide hormone, its levels are decreased in obesity and its receptors are expressed in the hypothalamus, the pituitary gland, and the testis. Adropin association to total testosterone in obese men is not detected yet. This study tries to find out possible associations between serum levels of adropin, adiponectin, total testosterone, and lipid profile in obese men.

**Methods:**

Serum levels of adropin, adiponectin, total testosterone, and lipid profile parameters were measured in 43 obese men and 40 age-matched normal-weight men.

**Results:**

Adropin, adiponectin, and testosterone levels were significantly lower in obese men versus normal-weight men. In all participants, positive correlations between adropin, adiponectin, and total testosterone were detected. Adropin is considered a predictor risk factor for testosterone.

**Conclusions:**

This study suggests a possible causal relationship between adropin and total testosterone which needs further investigation.

**Trial registration:**

Clincialtrials.gov NCT03724825, registered October 30th, 2018.

## Background

Obesity is a worldwide increasing problem, which is caused mainly by a positive energy balance in the form of increased energy intake than energy expenditure [1]. Obesity is linked to many health consequences as dyslipidaemia, diabetes mellitus, hypertension, and low testosterone levels in men [2]. Low testosterone levels not only predispose to infertility but also are considered biomarkers for mortality and morbidity in men [3]. Decreased muscle mass and bone mineral density [4], increased insulin resistance, risk of diabetes mellitus, low high-density lipoprotein cholesterol (HDL-c) levels, high triglycerides (TG) levels, hypertension, atherosclerosis, and myocardial infarction all are related to low testosterone levels [5]. Obesity can alter testosterone levels by many mechanisms. However, the exact mechanism is highly complex [6]. Adipokines, cytokines, and inflammation play a role in decreasing testosterone levels in obesity [7]. High aromatase enzyme expression that converts testosterone to oestrogen also affects testosterone levels by negative inhibition on the hypothalamic-pituitary-gonadal (HPG) axis [8]. Also, obesity-associated oxidative stress can injure testicular tissues and decrease testosterone levels [9].

Adropin, a 76 amino acid peptide hormone, was first discovered by Kumar et al [10] in obese mice during the microarray analysis of liver gene expression. It is encoded by the energy homeostasis-associated (Enho) gene [10], which is expressed in various organs such as the brain, liver, kidney, heart, pancreas, and human umbilical vein [11]. The orphan G protein-coupled receptor (GPR 19) is present in several tissues as the hypothalamic nuclei, the cerebellum, the testis, the heart, the liver, and the kidneys [12]. It is suggested to be the possible site of adropin action [13]. Adropin plays a role in energy homeostasis through the control of glucose and fatty acid metabolism [14]. It also has an anti-inflammatory [15] and an anti-oxidant effect [16]. In addition, it is described as a membrane-bound protein that binds with NB-3 and activates the Notch1 signaling pathway in the brain which is required for normal cerebellar development [17].

Adropin was found to decrease in obese subjects compared to overweight and normal-weight subjects, and its level negatively correlated with body mass index (BMI) [18]. Intra-peritoneal adropin administration to diet-induced obese (DIO) rats reduced total cholesterol (TC), TG, and low-density lipoprotein cholesterol (LDL-c) levels and increased HDL-c levels [15].

Lower adiponectin levels were detected in obese subjects than in the normal-weight subjects [19–21]. Circulating adiponectin levels showed a positive correlation with HDL-c and a negative correlation with TG and very-low-density lipoprotein (VLDL) levels [22]. Adiponectin can regulate testosterone secretion possibly through acting on the HPG axis [23]. Adiponectin inhibits gonadotrophin-releasing hormone (GnRH) release from the hypothalamus and luteinizing hormone (LH) release from the pituitary gland [24]. In addition, it can directly affect testosterone levels by acting on the testis, as adiponectin receptor 1 (Adipo R1) and adiponectin receptor 2 (Adipo R2) were identified in the testis [25].

Several mechanisms can affect testosterone levels, Adropin hormone may play a role however, up to our knowledge no previous literature clarified the relation between adropin and testosterone levels in normal-weight and obese men. This study aims to find out possible relations between adropin, adiponectin, lipid profile parameters, and total testosterone levels in normal-weight and obese men.

## Methods

This case-control study aims to find possible associations between total testosterone, adropin, adiponectin, and lipid profile parameters. 43 obese Egyptian men were included in this study (BMI ≥ 30 kg/m2) and age-matched 40 normal-weight men (BMI ≥18.5 and ≤ 25 kg/m2). The sample size was calculated by G Power 3 software [26] to detect an effect size of 0.2 in the mean adropin and/or adiponectin levels, with an α error probability of 0.05 and 80% power.

Included in this study were men aged from 18 years to 50 years. Subjects with known diabetes mellitus, hypertension, heart failure, kidney diseases, liver diseases, smokers, and subjects with a history of obstructive sleep apnea were excluded.

Blood samples were collected from obese and normal-weight men after fasting for 8-12 hours. Blood was centrifuged at 3000 rpm for 15 minutes to separate serum. The clear, non-haemolysed supernatant was separated and stored at -20°C until analysis. Total serum testosterone levels were measured by VIDAS Testosterone kits (Catalog No. 414320) purchased from Biomerieux, SA, France by Enzyme-linked fluorescent assay (ELFA) technique with intra-assay: coefficient of variability (CV)< 8% and inter-assay: CV≤ 12% for samples with a concentration between 0.3 ng/mL and 3 ng/mL and CV ≤ 10% for samples with a concentration greater than 3 ng/mL. Enzyme-Linked immunosorbent assay (ELISA) technique using kits purchased from SinoGeneClon Biotech Co., Ltd., China. were used to measure adropin (Catalog No. SG-11595) with a sensitivity: 0.5 ng/ml, intra-assay: CV< 8%, and inter-assay: CV<10%, and serum adiponectin levels (Catalog No. SG-10421) with a sensitivity: 5 ng/ml, intra-assay: CV< 8%, and inter-assay: CV <10%. Fasting serum blood glucose, TC, TG, and HDL-c levels were determined by the enzymatic colorimetric method by kits purchased from Biotecnica instruments S.p.A, Italy. The equation described by Friedewald et al [27] was used to determine serum LDL-c levels.

### Statistical analysis

Values were analysed using Statistical Package for Social Sciences (SPSS) version 26. The collected data were subjected to the Shapiro-Wilk test for testing normality. If the distribution was normal, the data were expressed as mean ± standard deviation, an independent t test, and Pearson correlation tests were used; however, if the distribution was not normal, the data were expressed as median and interquartile range, Mann–Whitney U test and Spearman correlation tests were applied.

## Results

Serum levels of TC, TG, and LDL-c were significantly higher, while HDL-c levels were significantly lower in obese when compared to normal-weight men (*P* < 0.05 for all). Significantly lower total testosterone, adropin, and adiponectin levels were detected in obese compared to normal-weight men (*P* < 0.05 for all) (Table 1).

**Table 1:** Fasting blood glucose, lipid profile, total testosterone, adropin, and adiponectin in normal-weight and obese men

Measured parameters	Normal-weight (*n*=40)	Obese (*n*=43)	Significance *P* value
Fasting blood glucose (mg/dl)	84.11± 16.45	89.09 ± 18.44	> 0.05
TC (mg/dl)	148.60 ± 24.53	199.58 ± 43.46	< 0.001
TG (mg/dl)	68.52 ± 23.65	147.28 ±101.32	< 0.001
HDL-c (mg/dl)	41.51 ± 11.06	35.58 ± 6.14	< 0.001
LDL-c (mg/dl)	93.62 ± 25.52	134.53 ± 33.32	< 0.001
Total testosterone (ng/ml)	6.48 ± 1.54	4.36 ± 1.44	< 0.001
Adropin (ng/ml)	25.45 (10.88)	9.60 (4.50)	< 0.001
Adiponectin (ng/ml)	1751.45 (1108.90)	901.90 (352.40)	< 0.001

Data were expresses as mean ± SD or median (interquartile range); P: significance of obese versus normal-weight group; A *P*-value < 0.05 is considered significant

*n*= number of subjects/group, *TC* Total cholesterol, *TG* Triglycerides, *HDL-c* High-density-lipoprotein cholesterol, *LDL-c* Low-density-lipoprotein cholesterol

Correlation analysis in normal-weight and obese men detected that BMI showed significant negative correlations with adropin, adiponectin, and total testosterone levels (*P* < 0.05 for all). Adropin had significant negative correlations with TC, TG, and LDL-c levels (*P* < 0.05 for all) but had a significant positive correlation with HDL-c levels (P < 0.05). Adiponectin showed significant negative correlations with TC, TG, and LDL-c levels (*P* < 0.05 for all), and a significant positive correlation with HDL-c levels (*P*< 0.05). Total testosterone was negatively correlated with TC, TG, and LDL-c levels (*P* < 0.05 for all), but positively correlated with HDL-c levels (*P* < 0.001) (Table 2).

**Table 2:** Correlations between adropin, adiponectin, total testosterone, and lipid profile

	Adropin (ng/ml)		Adiponectin (ng/ml)		Total testosterone (ng/ml)	
	r	*P*	r	*P*	r	*P*
BMI (kg/m^2^)	- 0.661	< 0.001	-0.562	< 0.001	-0.553	< 0.001
TC (mg/dl)	- 0.475	< 0.001	- 0.357	< 0.001	-0.266	0.015
TG (mg/dl)	-0.553	< 0.001	- 0.417	< 0.001	-0.374	< 0.001
LDL-c (mg/dl)	- 0.285	0.009	- 0.216	0.049	-0.260	0.018
HDL-c (mg/dl)	0.265	0.015	0.286	0.009	0.433	<0.001

r: correlation coefficient; *P*: significance level; *P*-value < 0.05 is considered significant

*BMI* Body mass index, *TC* Total cholesterol, *TG* Triglycerides, *HDL-c* High-density-lipoprotein cholesterol, *LDL-c* Low-density-lipoprotein cholesterol

**Fig. 1:**
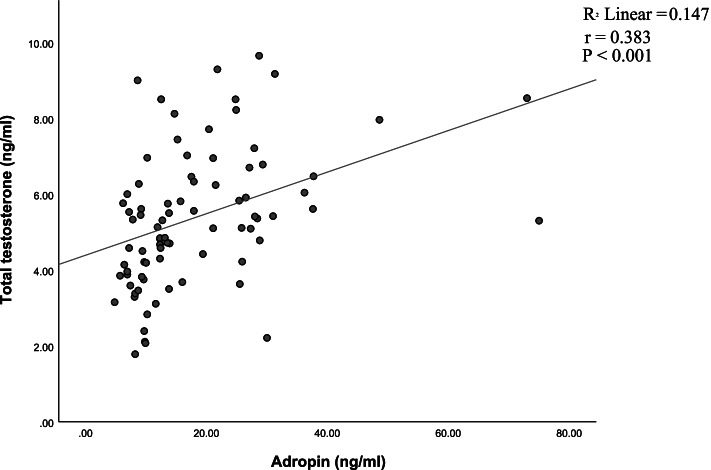
Correlation between adropin and total testosterone levels in normal-weight and obese men

**Fig. 2:**
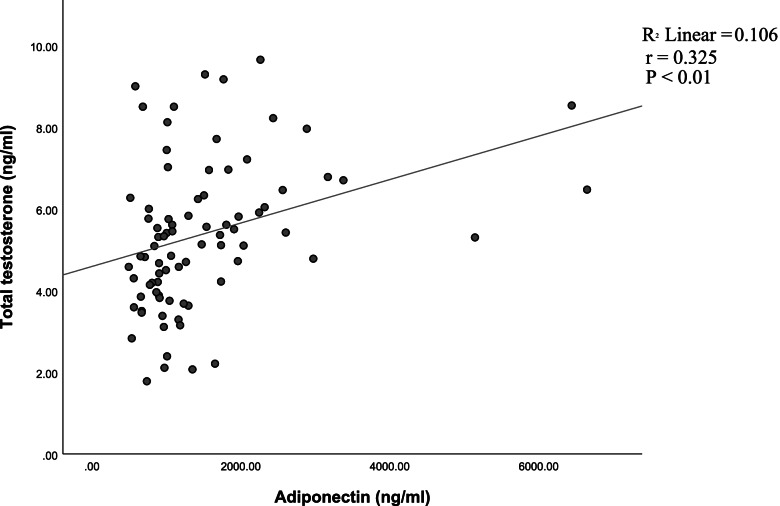
Correlation between adiponectin and total testosterone levels in normal-weight and obese men

**Fig. 3:**
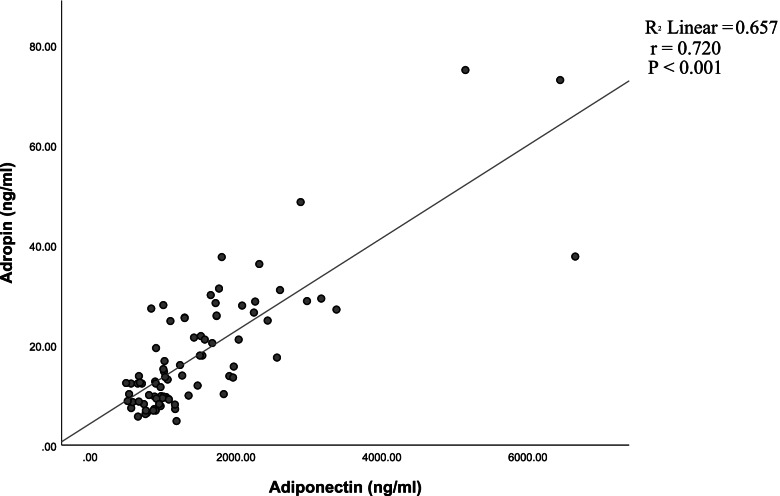
Correlation between adiponectin and adropin levels in normal-weight and obese men

Significant positive correlation between adropin and total testosterone levels was determined (*r* = 0.383, *P* < 0.001) (Fig. 1). Adiponectin correlated positively with total testosterone levels (*r* = 0.325, *P* < 0.01) (Fig. 2) and positively with adropin (*r* = 0.720, *P* < 0.001) (Fig. 3).

By multivariate stepwise regression analysis, the independent predictors of serum testosterone level according to B levels in order are HDL-c (B **=** 0.065, *P* < 0.01), adropin (B = 0.034, *P* < 0.05), and TG (B = -0.005, *P* < 0.05) (Table 3), while adiponectin, TC and LDL-c showed insignificant association with testosterone (*P* > 0.05 for all) (not shown on table).

**Table 3:** Multivariate stepwise regression analysis showing significant predictors of total testosterone

Variables	B ± SE	95% CI for B	*P*
HDL-c (mg/dl)	0.065 ± 0.019	0.028 – 0.103	0.001
Adropin (ng/ml)	0.034 ± 0.014	0.006 – 0.063	0.019
TG (mg/dl)	-0.005 ± 0.002	-0.009 – - 0.0003	0.036

B: B coefficient; *SE* Standard error, *95% CI* 95% confidence interval; R square for the model is 0.317; P: significance. P is significant if < 0.05; *HDL-c* High-density-lipoprotein cholesterol, *TG* Triglycerides

The independent predictors of serum adropin level by multivariate stepwise regression analysis, according to B levels in order are adiponectin (B **=** 0.009, *P* < 0.001), and TG (B = - 0.022, *P* < 0.05) (Table 4), while testosterone, TC and LDL-c showed insignificant association with adropin (*P* > 0.05 for all) (not shown on table).

**Table 4:** Multivariate stepwise regression analysis showing significant predictors of adropin

Variables	B ± SE	95% CI for B	*P*
Adiponectin (ng/ml)	0.009 ± 0.001	0.007 – 0.010	<0.001
TG (mg/dl)	-0.022 ± 0.010	- 0.042 – - 0.003	0.027

*B* B coefficient; *SE* Standard error; *95% CI* 95% confidence interval; R square for model is 0.678; *P*: significance; *P* is significant if < 0.05; *TG* Triglycerides

The independent predictors of serum adiponectin level is adropin (B **=** 70.838, *P* < 0.001) (Table 5), while testosterone, TC, TG and LDL-c showed insignificant association with adiponectin (*P* > 0.05 for all) (not shown on table).

**Table 5:** Multivariate stepwise regression analysis showing significant predictors of adiponectin

Variable	B ± SE	95% CI for B	*P*
Adropin (ng/ml)	70.838 ± 5.683	59.532 – 82.145	<0.001

*B* B coefficient, *SE* Standard error; *95% CI* 95% confidence interval; R square for model is 0.657; *P*: significance; *P* is significant if < 0.05.

## Discussion

Obesity is associated with low testosterone levels in men [28]. The relationship of adropin to total testosterone in normal-weight and obese men requires further investigation. The current study attempts to determine changes in adropin levels and their possible relationship to testosterone, adiponectin, and lipid profile.

Total testosterone levels in obese men were significantly lower than in normal-weight men, and they were negatively associated with BMI. Several previous studies found low testosterone levels in obese men [29–31].. Obesity, with its associated insulin resistance and high TG levels, has been linked to lower testosterone levels [32]. Decreased adiponectin secretion from adipose tissue may also play a role [7]. High aromatase enzyme expression, which converts testosterone to oestrogen, lowers testosterone levels via HPG axis negative inhibition [8]. Furthermore, obesity-related increases in TNF- can directly inhibit LH secretion [33].

This study found a significant positive correlation between adropin and total testosterone. Furthermore, stepwise regression analysis showed that HDL-c, adropin, and TG are significant predictors of testosterone levels. In line with this, Fernández-Miró et al [5] discovered that low testosterone levels were associated with low HDL-c and high TG levels in men, and Hagiuda et al [32] found that TG was a significant risk factor for decreased serum testosterone levels in men. To our knowledge, no previous study has described the relationship between adropin and testosterone in men.

Adropin may influence testosterone levels by affecting TG levels. Adropin administration to rats decreased TG synthesis [34], implying that lower adropin levels in obesity are associated with higher TG levels, which are considered a significant predictor of testosterone levels, as mentioned in this study and the Hagiuda et al [32] study.

Adropin administration decreased TNF- α levels in streptozotocin-induced type 2 diabetic rats [15]. Obesity is associated with low adropin levels and high TNF-α levels which inhibit LH production and decrease total testosterone levels [35]. Besides that, high oxidative stress levels associated with low adropin [16] are linked to low testosterone levels [36].

Adropin has been identified as a predictor risk factor for adiponectin levels in this study, which is supported by Kumar et al [10] who demonstrated that adropin injection in mice increased serum adiponectin levels. Adropin directly increased adiponectin mRNA expression in rat adipocytes [37]. Besides that, adropin administration in streptozotocin-induced type 2 diabetic rats decreased TNF-α levels [15]. Obesity-associated low adropin levels are related to high TNF-α levels, which act on adipocytes decreasing adiponectin mRNA stability and expression, resulting in decreased adiponectin formation [38]. Thus low adropin levels associated with obesity could modulate testosterone levels by decreasing adiponectin levels.

Adiponectin could influence testosterone secretion by acting on the HPG axis [23]. It inhibits hypothalamic GnRH secretion as well as pituitary LH release [24]. Adiponectin receptors were discovered in the testis [25], and its administration to rats reduced testosterone levels in the testis [39]. This study revealed a positive association between adiponectin and testosterone. Thomas et al [40] found a positive correlation between adiponectin and testosterone in normal-weight and obese adult men. Riestra et al [41] found a negative but not significant correlation between adiponectin and testosterone in normal-weight adolescents. Furthermore, Song et al [42] found no significant correlation between adiponectin and testosterone in normal-weight old men. This could be explained by differences in testosterone levels across age groups, which affect adiponectin levels.

In this study, obese men had significantly lower adiponectin levels than normal-weight men, which is consistent with Thanakun et al [20] and Li et al [21], who found lower adiponectin levels in obese subjects. Obesity-associated low adiponectin levels could reduce adiponectin inhibition on GnRH and LH secretion, increasing testosterone levels [24]. However, adiponectin can affect testosterone in other ways, as illustrated below: Obesity-related low adiponectin levels raise TG levels by decreasing TG catabolism [22], which is a significant risk factor for low testosterone levels in men [32]. TNF-α production in the liver is reduced by adiponectin [43]. As a result, lower adiponectin levels in obesity may be associated with increased TNF-α production, which directly inhibits pituitary LH secretion [35]. Low adiponectin levels have been linked to high levels of oxidative stress [44], which may further reduce testosterone levels [36]. Although low adiponectin levels can increase testosterone levels when acting directly on the hypothalamus, pituitary, and testis, it can also decrease testosterone levels by increasing TG levels, TNF-α, and oxidative stress levels. The sum of these mechanisms could be the net effect of adiponectin on testosterone.

Another way adropin can affect testosterone is by acting directly on the hypothalamus, affecting GnRH secretion. Loewen and Ferguson [45] discovered that the majority of cells in the paraventricular nucleus responded to adropin bath application via depolarization or hyperpolarization. More studies are needed to determine the precise effect of adropin on GnRH secretion. Concerning the effect of adropin on the pituitary gland and the testis, Hoffmeister-Ullerich et al [12] discovered GPR19 expression in the pituitary gland and testis of mice, implying a possible adropin role in the pituitary gland and the testis. The exact effect of adropin on LH and testosterone secretion requires further research.

This study has several limitations that must be addressed. This study did not rule out hypothyroidism or hyperthyroidism, both of which can affect lipid profile levels. Furthermore, adiponectin, adropin, and testosterone levels may be affected by age. Participants in this study ranged in age from 18 to 50 years old; smaller age groups may be required to test the effects of age on these parameters. Future research is needed to confirm the effect of adropin on testosterone levels, which could be accomplished by injecting adropin into rats or culturing testicular, pituitary, or hypothalamic tissues with adropin, both of which were unavailable to the authors at the time of the study.

## Conclusion

Many factors contribute to obesity-related low testosterone levels. Adropin hormone was discovered to be a predictor risk factor of testosterone in this study, implying that adropin may play a role in influencing testosterone levels. This could be accomplished by affecting TG and adiponectin levels. More research is needed to determine exactly how adropin affects testosterone levels.

## Data Availability

The datasets used and/or analysed during the current study are available from the corresponding author on reasonable request.

## Abbreviations

HDL-c: High-density lipoprotein cholesterol
TG: Triglycerides
HPG: Hypothalamic-pituitary-gonadal
Enho: Energy homeostasis-associated
GPR: G protein-coupled receptor
BMI: Body mass index
DIO: Diet-induced obese
TC: Total cholesterol
LDL-c: Low-density lipoprotein cholesterol
VLDL: Very-low-density lipoprotein
GnRH: Gonadotrophin-releasing hormone
LH: Luteinizing hormone
Adipo R1: Adiponectin receptor 1
Adipo R2: Adiponectin receptor 2
ELISA: Enzyme-Linked immunosorbent assay
(CV): Coefficient of variability

## References

[1] Blüher M (2019). Obesity: global epidemiology and pathogenesis. Nat Rev Endocrinol.

[2] Hruby A, Manson JE, Qi L, Malik VS, Rimm EB, Sun Q (2016). Determinants and consequences of obesity. Am J Public Health.

[3] Morgentaler A, Miner MM, Caliber M, Guay AT, Khera M, Traish AM (2015). Testosterone therapy and cardiovascular risk: advances and controversies. Mayo Clin Proc..

[4] Cunningham GR, Stephens-Shields AJ, Rosen RC, Wang C, Ellenberg SS, Matsumoto AM (2015). Association of sex hormones with sexual function, vitality, and physical function of symptomatic older men with low testosterone levels at baseline in the testosterone trials. J Clin Endocrinol Metab.

[5] Fernández-Miró M, Chillarón JJ, Pedro-Botet J (2016). Testosterone deficiency, metabolic syndrome and diabetes mellitus. Medicina Clínica (English Edition).

[6] Fernandez CJ, Chacko EC, Pappachan JM (2019). Male obesity-related secondary hypogonadism pathophysiology, clinical implications and management. Eur Endocrinol.

[7] Tsatsanis C, Dermitzaki E, Avgoustinaki P, Malliaraki N, Mytaras V, Margioris ANJH (2015). The impact of adipose tissue-derived factors on the hypothalamic-pituitary-gonadal (HPG) axis. Hormones.

[8] Kelly D, Jones T (2015). Testosterone and obesity. Obes Rev.

[9] Zhao J, Zhai L, Liu Z, Wu S, Xu L. longevity c. Leptin level and oxidative stress contribute to obesity-induced low testosterone in murine testicular tissue. Oxid Med Cell Longev. 2014;2014:1–14. 10.1155/2014/190945 PMC4009340 24829619

[10] Kumar KG, Trevaskis JL, Lam DD, Sutton GM, Koza RA, Chouljenko VN (2008). Identification of adropin as a secreted factor linking dietary macronutrient intake with energy homeostasis and lipid metabolism. Cell Metab.

[11] Shahjouei S, Ansari S, Pourmotabbed T, Zand R (2016). Potential roles of adropin in central nervous system: review of current literature. Front Mol Biosci.

[12] Hoffmeister-Ullerich SA, Süsens U, Schaller HC (2004). research t. The orphan G-protein-coupled receptor GPR19 is expressed predominantly in neuronal cells during mouse embryogenesis. Cell Tissue Res.

[13] Thapa D, Stoner MW, Zhang M, Xie B, Manning JR, Guimaraes D (2018). Adropin regulates pyruvate dehydrogenase in cardiac cells via a novel GPCR-MAPK-PDK4 signaling pathway. Redox Biol.

[14] Gao S, McMillan RP, Jacas J, Zhu Q, Li X, Kumar GK (2014). Regulation of substrate oxidation preferences in muscle by the peptide hormone adropin. Diabetes.

[15] Akcilar R, Kocak F, Simsek H, Akcilar A, Bayat Z, Ece E (2016). Antidiabetic and hypolipidemic effects of adropinin streoptozotocin-induced type 2 diabetic rats. Bratislavske lekarske listy.

[16] Chen X, Xue H, Fang W, Chen K, Chen S, Yang W (2019). Adropin protects against liver injury in nonalcoholic steatohepatitis via the Nrf2 mediated antioxidant capacity. Redox Biol.

[17] Wong C-M, Wang Y, Lee JTH, Huang Z, Wu D, Xu A (2014). Adropin is a brain membrane-bound protein regulating physical activity via the NB-3/Notch signaling pathway in mice. J Biol Chem.

[18] Zang H, Jiang F, Cheng X, Xu H, Hu X (2018). Serum adropin levels are decreased in Chinese type 2 diabetic patients and negatively correlated with body mass index. Endocrine J.

[19] Vargas R, Ryder E, Diez-Ewald M, Mosquera J, Durán A, Valero N (2016). Increased C-reactive protein and decreased Interleukin-2 content in serum from obese individuals with or without insulin resistance: Associations with leukocyte count and insulin and adiponectin content. Diabetes Metab Syndr.

[20] Thanakun S, Pornprasertsuk-Damrongsri S (2017). Izumi YJOd. Increased oral inflammation, leukocytes, and leptin, and lower adiponectin in overweight or obesity. Oral Diseases..

[21] Li T, Yang L, Zhao S, Zhang S, Research C (2018). Correlation between apolipoprotein M and inflammatory factors in obese patients. Medical Sci Monit.

[22] Christou G, Kiortsis D (2013). Adiponectin and lipoprotein metabolism. Obesity Rev.

[23] Wen J-P, Lv W-S, Yang J, Nie A-F, Cheng X-B, Yang Y (2008). Globular adiponectin inhibits GnRH secretion from GT1-7 hypothalamic GnRH neurons by induction of hyperpolarization of membrane potential. Biochem Biophys Rescommun.

[24] Cheng X-B, Wen J-P, Yang J, Yang Y, Ning G, Li X-Y (2011). GnRH secretion is inhibited by Adiponectin through activation of AMP-activated protein kinase and extracellular signal regulated kinase. Endocrine.

[25] Caminos J, Nogueiras R, Gaytán F, Pineda R, Gonzalez C, Barreiro M (2008). Novel expression and direct effects of adiponectin in the rat testis. Endocrinology.

[26] Faul F, Erdfelder E, Lang A, Buchner AJBRM (2007). A flexible statistical power analysis program for the social, behavioral and biomedical sciences. Behav Res Methods.

[27] Friedewald WT, Levy RI, Fredrickson DS (1972). Estimation of the concentration of low-density lipoprotein cholesterol in plasma, without use of the preparative ultracentrifuge. Clin Chem.

[28] Rastrelli G, Carter EL, Ahern T, Finn JD, Antonio L, O'Neill TW (2015). Development of and recovery from secondary hypogonadism in aging men: prospective results from the EMAS. J Clin Endocrinol Metab.

[29] Eriksson J, Haring R, Grarup N, Vandenput L, Wallaschofski H, Lorentzen E (2017). Causal relationship between obesity and serum testosterone status in men: a bi-directional mendelian randomization analysis. Plos One.

[30] Canguven O, Talib R, El Ansari W, Yassin DJ, Salman M, Al-Ansari AJA (2017). Testosterone therapy has positive effects on anthropometric measures, metabolic syndrome components (obesity, lipidv profile, Diabetes Mellitus control), blood indices, liver enzymes, and prostate health indicators in elderly hypogonadal men. Andrologia.

[31] Amjad S, Baig M, Zahid N, Tariq S, Rehman RJA (2019). Association between leptin, obesity, hormonal interplay and male infertility. Andrologia.

[32] Hagiuda J, Ishikawa H, Furuuchi T, Hanawa Y, Marumo KJA (2014). Relationship between dyslipidaemia and semen quality and serum sex hormone levels: an infertility study of 167 Japanese patients. Andrologia.

[33] Huang G, Yuan M, Zhang J, Li J, Gong D, Li Y (2016). IL-6 mediates differentiation disorder during spermatogenesis in obesity-associated inflammation by affecting the expression of Zfp637 through the SOCS3/STAT3 pathway. Sci Rep.

[34] Gao S, Ghoshal S, Zhang L, Stevens JR, McCommis KS, Finck BN (2019). The peptide hormone adropin regulates signal transduction pathways controlling hepatic glucose metabolism in a mouse model of diet-induced obesity. J Biol Chem.

[35] Lamm S, Chidakel A, Bansal R (2016). Obesity and hypogonadism. Urol Clin.

[36] Abbasihormozi S, Babapour V (2019). Stress hormone and oxidative stress biomarkers link obesity and diabetes with reduced fertility potential. Cell J (Yakhteh).

[37] Jasaszwili M, Pruszyńska-Oszmałek E, Wojciechowicz T, Strowski MZ, Nowak KW, Skrzypski M (2021). Adropin Slightly Modulates Lipolysis, Lipogenesis and Expression of Adipokines but Not Glucose Uptake in Rodent Adipocytes. Genes.

[38] Wang Y, Wang H, Hegde V, Dubuisson O, Gao Z, Dhurandhar NV (2013). Interplay of pro-and anti-inflammatory cytokines to determine lipid accretion in adipocytes. Int J Obes.

[39] Pfaehler A, Nanjappa MK, Coleman ES, Mansour M, Wanders D, Plaisance EP (2012). Regulation of adiponectin secretion by soy isoflavones has implication for endocrine function of the testis. Toxicol Lett.

[40] Thomas S, Kratzsch D, Schaab M, Scholz M, Grunewald S, Thiery J (2013). Seminal plasma adipokine levels are correlated with functional characteristics of spermatozoa. Fertil Steril.

[41] Riestra P, Garcia-Anguita A, Ortega L, Garcés C (2013). Relationship of adiponectin with sex hormone levels in adolescents. Horm Res Paediatr.

[42] Song HJ, Oh S, Quan S, Ryu O-H, Jeong J-Y, Hong K-S (2014). Gender differences in adiponectin levels and body composition in older adults: Hallym aging study. BMC Geriatr.

[43] Xu A, Wang Y, Keshaw H, Xu LY, Lam KS, Cooper G (2003). The fat-derived hormone adiponectin alleviates alcoholic and nonalcoholic fatty liver diseases in mice. J Clin Investig.

[44] Frühbeck G, Catalán V, Rodríguez A, Ramírez B, Becerril S, Salvador J (2017). Involvement of the leptin-adiponectin axis in inflammation and oxidative stress in the metabolic syndrome. Sci Rep.

[45] Loewen SP, Ferguson AV (2017). Integrative, Physiology C. Adropin acts in the rat Paraventricular nucleus to influence neuronal excitability. Am J Physiol Regul Integr Comp Physiol.

